# Lichenoid drug reaction after ipilimumab/nivolumab combination therapy: A case report

**DOI:** 10.1177/2050313X231213927

**Published:** 2023-12-20

**Authors:** Zonía Robenne Moore, Dalit Zajdman-Faitelson, Arely Tamariz Campillo, Diana Karen Brito Bustillos, Sonia Toussaint-Caire, Cristina Berumen-Glinz

**Affiliations:** 1 University of Pennsylvania Perelman School of Medicine, Philadelphia, PA, USA; 2 Dermatology Department, Manuel Gea González General Hospital, Tlalpan, CDMX, Mexico; 3 Dermatopathology Department, Manuel Gea González General Hospital, Tlalpan, CDMX, Mexico

**Keywords:** Bullous lichenoid drug reaction, ipilimumab, nivolumab, combination therapy, mesothelioma

## Abstract

Nivolumab (PD-1 inhibitor) and ipilumumab (CTLA-4 inhibitor) are recently approved checkpoint inhibitors for treatment of non-small cell lung cancer. Immune-related adverse events related to the usage of checkpoint inhibitors are growing with their popularity. We present the case of a patient in combination treatment of nivolumab and ipilimumab who developed a lichenoid drug reaction, notable because it worsened to a bullous lichenoid drug reaction. Treatment with prednisone and withdrawal of checkpoint inhibitors aided in clinical resolution. Initial presentation of a lichenoid reaction that progressed to a bullous, desquamated presentation indicates the possibility of the prodromal rash progressing to a Stevens-Johnson Syndrome-like dermatosis. When dermatologists are consulted for rashes developed during checkpoint-inhibitor therapy, they should be aware that early treatment may prevent progression to bullae formation and desquamation and develop their treatment plans with this in mind.

## Background

Checkpoint inhibitors are gaining popularity in the treatment of skin and lung cancers. Nivolumab is a PD-1 inhibitor that was first approved to treat metastatic melanoma in 2014 by the Food and Drug Administration. Ipilimumab is a CTLA-4 inhibitor approved in March 2011 to treat late-stage unresectable melanoma. Both drugs have since been approved for treatment of non-small cell lung cancer (NSCLC).^1,2^

Mesothelioma is a lung cancer most frequently caused by asbestos exposure; usage of checkpoint inhibitors as treatment is being established.^1,2^ Immune-related adverse events (ir-AE) are more frequent as checkpoint inhibitor popularity grows. Most ir-AEs are low grade: a review, including 5,744 NSCLC patients from 23 studies treated with anti-PD-1, found an incidence of ir-AEs of 64% with anti-PD-1 agents (14% grade ⩾3).^1,2^ These grade 3 reactions exist on a spectrum from lichenoid drug reaction to Stevens-Johnson Syndrome/Toxic Epidermal Necrolysis (SJS/TEN). Here we present a case of a lichenoid dermatitis from ipilimumab/nivolumab combination therapy prevented from evolving into an SJS-like dermatosis through early management.

## Case presentation

We present the case of a 68-year-old man diagnosed with unresectable biphasic mesothelioma in February 2022, and underwent four cycles of chemotherapy with gemcitabine (410 mg) and cisplatin (57 mg). Due to disease progression, treatment changed to an immune checkpoint inhibitor. In August 2022, he received two cycles of combination ipilimumab (63 mg)/nivolumab (180 mg) treatment.

Two weeks after his last nivolumab/ipilimumab dose, he developed a generalized scaly erythematous eruption with thick scale and associated burning sensation, pruritus, chills, and fatigue. Due to erythroderma and acute skin failure, treatment was started with prednisone 60 mg daily (1 mg/kg) and a punch biopsy was performed.

**Figure 1.**
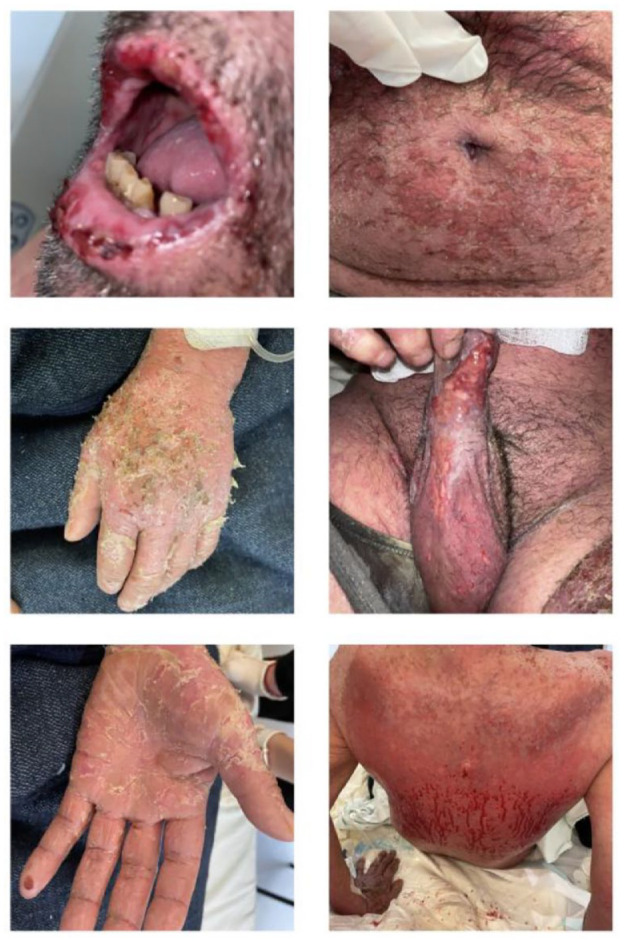
Initial presentation of the patient’s dermatosis on day 1 of hospital admission. Flaccid bullae had burst, leaving exulcerations on the patient’s skin.

One week later, the patient had worsened. He experienced generalized 8/10 pain, chills, fatigue, and pain with eating. Cutaneous examination revealed additional poorly delimited erosions on his back, abdomen, penis, scrotum, and inguinal and intergluteal folds that affected affecting approximately 20% of body surface area (according to Palmer method of estimation; see Figure 1). He also presented scaly erythematous plaques and fissures on his hands and elbows with poorly defined ulcerations on his vermilion lip, buccal, and palatine mucosa. The patient was admitted to *Instituto Nacional de Cancerología*, for treatment with IV methylprednisolone 70 mg/day, Kaomycin mouth washes every 8 h, and vaseline-bandage changes on denuded skin every 48 h.

Two days later, the erosions on his back and his intraoral ulcers were partially re-epithelialized, and associated symptoms had improved. He was able to stand and eat, and much of the thick scale had fallen off. Four days later, the patient continued with improvements on his extremities and thorax, with approximately 80% re-epithelization. He was discharged with continued prednisone.

**Figure 2.**
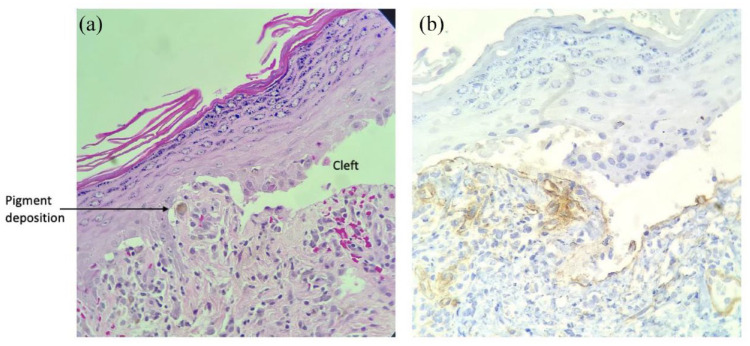
Histopathology results. A, 20×, Hypergranulosis and the beginning of the subepidermal cleft with pigment deposition, a chronic inflammatory infiltrate of histiocytes and lymphocytes along with extravasation of erythrocytes. B, 40×, Immunohistochemical stain for type IV collagen showing the basement membrane at the bottom of the cleft.

**Figure 3.**
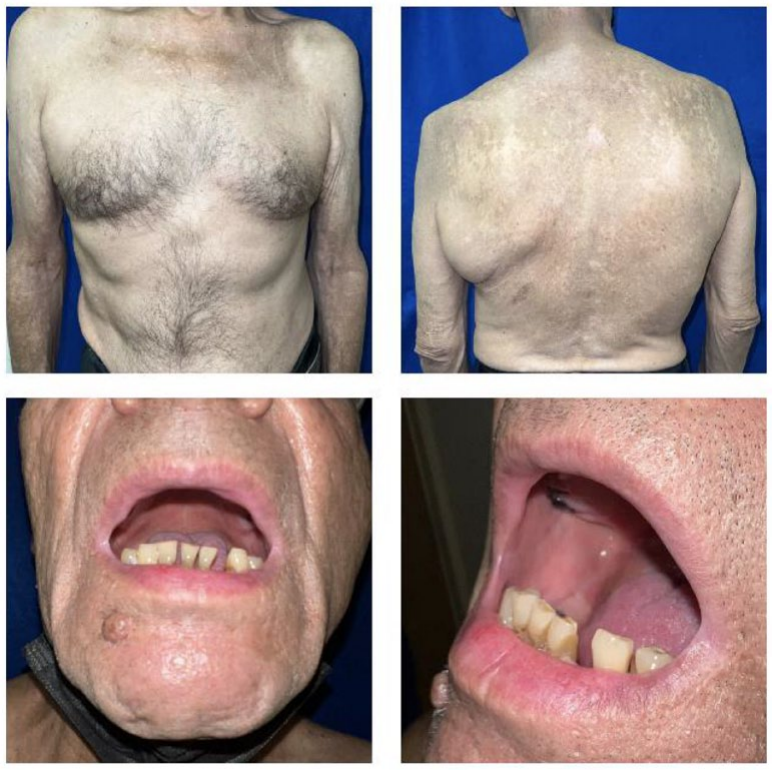
Patient recovery at the end of therapy. Photos taken 6 months after hospital discharge.

On his first follow-up visit he presented complete re-epithelialization and total symptomatic improvement. Histopathological results were obtained by then, showing an orthokeratotic stratum corneum with focal parakeratosis, hypergranulosis, necrotic suprabasal keratinocytes, and vacuolar damage to the dermal–epidermal interface. A band of lymphocyte and histiocyte infiltration with erythrocyte extravasation was observed beneath a subepidermal separation. An immunostain for collagen IV confirmed the presence of that collagen at the bottom of the separation. These findings are presented in Figure 2. Immune checkpoint-inhibitor therapy was discontinued. He received third and fourth line of therapy with pemetrexed and vinorelbine, respectively, with disease progression. His skin re-epithelialization can be seen in Figure 3.

## Discussion

This case presentation is unusual due to the bullous presentation of checkpoint-inhibitor-induced lichenoid reaction. The physiology behind this presentation is unknown, but our histopathological and clinical results support a hypothesis of a lichenoid drug reaction with extensive epidermal detachment producing a bullous lichenoid reaction or Stevens-Johnson Syndrome-like (SJS-like) reaction. In Figure 2, we see epidermal detachment and a subepidermal cleft, which supports the above theory. Collagen IV is found beneath the epidermis in bullous lichen planus and in pemphigoid reactions, which does not distinguish the disorders.^3^ Attempts were made to obtain autoantibody titers; however, (Dsg) 1, anti-DSG3, and anti-BP-180 autoantibody titers are unavailable at the time of writing in our zone.^3^ In this case, early intervention with prednisone 1 mg/kg and checkpoint withdrawal prevented full SJS-like development. The rapid response to therapy makes a diagnosis of a bullous lichenoid drug reaction more likely than a pemphigoid reaction.

The initial liquenoid-like reaction worsened to flaccid bullae development before resolving. In their systematic review of 29 cases of anti-PD1 therapy-associated bullous disorders, Zhao et al. found six cases of SJS/TEN/Erythema Multiforme (EM) reactions with an atypical prodrome and an interface dermatitis shown on biopsy prior to development of SJS/TEN/EM.^4^

Potts et al. report a case of a drug-induced lichen planus evolving into SJS in 2022. Their patient had “pruritic erythematous coalescing patches and plaques most severe on the forearms and lower legs. After a third dose of nivolumab, the rash worsened. Biopsy showed a non-specific lichenoid dermatosis. Nine months after starting nivolumab and six months after her last dose, the patient’s rash worsened over a number of weeks, with intense pruritus and hemorrhagic skin sloughing.”^1^ The clinical course in this case is quite similar to ours. We intervened earlier with a similar dosage of prednisone and stopped administration of nivolumab, preventing progression to SJS-like symptoms.

Enomoto et al. reported on a man in his 50s with advanced lung adenocarcinoma, treated with nivolumab every 2 weeks. Three days after his 10^th^ dose, the patient developed severe pain in his oral cavity. The patient did not respond to topical corticosteroid, and nivolumab was discontinued. One month later, the patient complained of dysphagia and severe sore throat, and was diagnosed with severe pharyngolaryngitis. Oral prednisone (1 mg/kg) was administered with resolution 3 weeks later. Histological analysis of biopsies taken after administration of topical corticosteroid showed a band-like infiltration of lymphocytes in the subepithelium with separation of the basal layer.^5^ This clinical course similarly supports our hypothesis, with the histological findings in Figure 2 showing our separation of the basement layer.

## Conclusion

Dermatologists should consider regularly following patients on checkpoint-inhibitor therapy as a part of preventative care. SJS-like reactions may progress from a lichenoid drug eruption through a mechanism of basement membrane exposition, placing bullous lichenoid eruptions on the same spectrum with SJS-like reactions of cutaneous cytotoxicity. Early intervention with 1 mg/kg prednisone and cessation of the checkpoint-inhibitor prevent the development of life-threatening grade 4 cutaneous adverse reactions. Careful clinical judgment and coordination with the treating oncologist needs to be used on whether to continue or withdraw the checkpoint inhibitor.
